# Comparison of HEp-2 and Vero Cell Responses Reveal Unique Proapoptotic Activities of the Herpes Simplex Virus Type 1 α0 Gene Transcript and Product

**DOI:** 10.3389/fmicb.2019.00998

**Published:** 2019-05-08

**Authors:** Marie L. Nguyen, Elisabeth Gennis, Kristen C. Pena, John A. Blaho

**Affiliations:** ^1^Department of Microbiology and Immunology, Des Moines University, Des Moines, IA, United States; ^2^Department of Microbiology, Mount Sinai School of Medicine, New York, NY, United States; ^3^NYC Regional Innovation Node, The City University of New York, New York, NY, United States

**Keywords:** apoptosis induction, α0 gene, ICP0 protein, HEp-2, Vero cells

## Abstract

Previous studies have provided evidence suggesting a role for apoptosis in the control of Herpes Simplex Virus 1 (HSV-1) latency. HSV-1 induces and then later blocks apoptosis in infected cells. The immediate early viral gene α0, which synthesizes the ICP0 protein, is necessary and sufficient for HSV-1-induced apoptosis in human epithelial (HEp-2) cells. While previous research showed that ICP0 protein synthesis is not necessary for HSV-1-induced apoptosis in infected HEp-2 cells, circumstantial evidence suggested that it might be needed in infected African green monkey kidney (Vero) cells. In this study, we determined the specific aspects of α0 needed to trigger apoptosis in these two cell types. HEp-2 cells transfected with α0 expressing plasmids that generated either full-length, truncated, or no detectable (multiple stop codons) ICP0 protein died through apoptosis. This indicates that ICP0 protein is not necessary for α0-induced apoptosis and that α0 mRNA alone has apoptotic induction properties in HEp-2 cells. We next investigated the primary structure of α0’s mRNA to better define its proapoptotic ability. Since α0 is one of the few HSV-1 genes that are spliced, we transfected cells with a plasmid expressing ICP0 from cDNA copy, pcDNAICP0. The cells transfected with pcDNAICP0 underwent apoptosis at a level equivalent to those transfected with the genomic copy of α0, which indicates that neither splicing events nor introns are required for the apoptotic function of α0 in HEp-2 cells. Next, we studied the ability of α0 to cause apoptosis in Vero cells. Since HSV-1-induced apoptosis in Vero cells requires protein synthesis early in infection, proteins synthesized with immediate early kinetics may facilitate apoptosis. Vero cells were transfected with plasmids producing either full-length ICP0 or ICP0 truncated at codon 212. Full-length ICP0, but not truncated ICP0, induced apoptosis in Vero cells. Together, these results suggest that α0 gene expression triggers apoptosis, but ICP0 protein is needed to facilitate apoptosis in Vero cells. In addition, ICP0’s facilitation activity may lie in its carboxyl-terminated domain. Thus, our results demonstrate that α0’s mRNA and protein possess proapoptotic properties. The requirement for ICP0 protein during HSV-dependent apoptosis appears to be cell type specific.

## Introduction

Herpes simplex virus 1 (HSV-1) is a large, enveloped DNA virus belonging to the *Herpesviridae* family. The most common clinical manifestation of HSV-1 infections is herpes labialis, commonly referred to as a cold sore. However, when the virus enters tissues outside of the oral epithelium, more serious disease outcomes occur. For example, HSV-1 infections of the cornea cause herpes simplex keratitis, which is the leading cause of infectious blindness in the United States (Liesegang et al., 1989). Furthermore, neonatal HSV infections often spread to the brain, causing life threatening encephalitis. The majority of neonate infections are the result of HSV transmission from maternal genital infections to newborn infects during childbirth. There has been an increase in genital HSV-1 infections in young women in the United States (Peñna et al., 2010). Therefore, insights in the HSV replication cycle have the potential to significantly impact human disease.

One of the defining features of the *Herpesviridae* family is the ability to form a latent state, from which reactivation events lead to subsequent virus replication and often clinical symptoms. HSV establishes a latent infection in the sensory neurons located at the sites of initial infection, e.g., trigeminal ganglia for oral HSV infections. Reactivation events throughout the lifespan of infected individuals lead to new rounds of lytic virus replication in adjacent epithelial tissues and recurrent herpetic lesions. There is evidence for cellular apoptotic events playing a role in controlling the latent and lytic states of the HSV life cycle (reviewed in Nguyen and Blaho, 2007).

Apoptosis is a form of programmed cell death that has been shown to be important for proper tissue development, prevention of tumors, and cellular responses to pathogens (reviewed in Koyama et al., 2003). Apoptotic cell death is distinguished from other forms of cell death by defined morphological and biochemical features displayed by the dying cells. These features include blebbing and alterations in the chemical makeup of the plasma membrane, condensation and eventual fragmentation of the chromosomal DNA, and loss of mitochondrial membrane potential (Kerr et al., 1972; Wyllie et al., 1980; Takano et al., 1991). One class of enzymes required for most forms of apoptotic cell death are the caspases (reviewed in Salvesen and Dixit, 1997; Villa et al., 1997). Caspases are synthesized as large inactive precursors, which are cleaved and form active tetramers during apoptosis. The caspases cleave their targets at specific peptide motifs containing aspartate residues. Caspase targets include caspases themselves and a variety of other cellular proteins, many which are involved in maintaining the structural or chemical integrity of the cell, e.g., lamin B, DFF/ICAD, and poly(ADP)ribose polymerase (PARP) (reviewed in Sanfilippo and Blaho, 2003).

There are two types of gene products abundantly produced during HSV latency. Both are transcripts from the R_L_ and surrounding regions of the genome. The long transcripts have been the most well-studied and are called the latency associated transcripts (LATs) (Stevens et al., 1987; Spivack and Fraser, 1988). More recently, microRNAs have also been found to be produced during latent infection (reviewed in Phelan et al., 2017). Infections with recombinant viruses harboring LAT deletions have been reported to yield reduced numbers of latently infected neurons compared to wild-type (Sawtell and Thompson, 1992; Thompson and Sawtell, 1997). Other studies reported a reduced ability for LAT mutants to reactivate from latently infected explants (Leib et al., 1989a). LATs have been shown to possess anti-apoptotic activities (Perng et al., 2000; Inman et al., 2001a; Ahmed et al., 2002; Jin et al., 2003). The anti-apoptotic regions of LATs have been correlated with the domains needed for latency (Perng et al., 2000; Inman et al., 2001a; Ahmed et al., 2002). Furthermore, replacement of LAT domains with other anti-apoptotic genes, such as the bovine herpesvirus 1 LR and Baculovirus cpIAP, restore LAT latency functions in rabbit and mouse HSV infection models (Perng et al., 2002; Jin et al., 2005, 2008). Finally, inducting apoptosis in latently infected trigeminal ganglia with dexamethasone accelerated their reactivation (Du et al., 2012). Thus, investigations into apoptosis in HSV-1 infected cells may provide insight into the establishment, maintenance, and/or reactivation from latency.

Previous studies have provided evidence that an intricate balance between apoptotic agonists and antagonists exists within herpes simplex virus-infected cells in both tissue culture and animal models (Nguyen and Blaho, 2009). Seven viral genes have been reported to possess apoptotic antagonistic properties. When these antagonists are not efficiently produced, such as infections in the presence of protein synthesis inhibitors or in the absence of key immediate early genes (vBSΔ27 infection) the apoptotic balance is upset, and the infected cells die through the intrinsic apoptotic pathway (Aubert et al., 2007).

**FIGURE 1:**
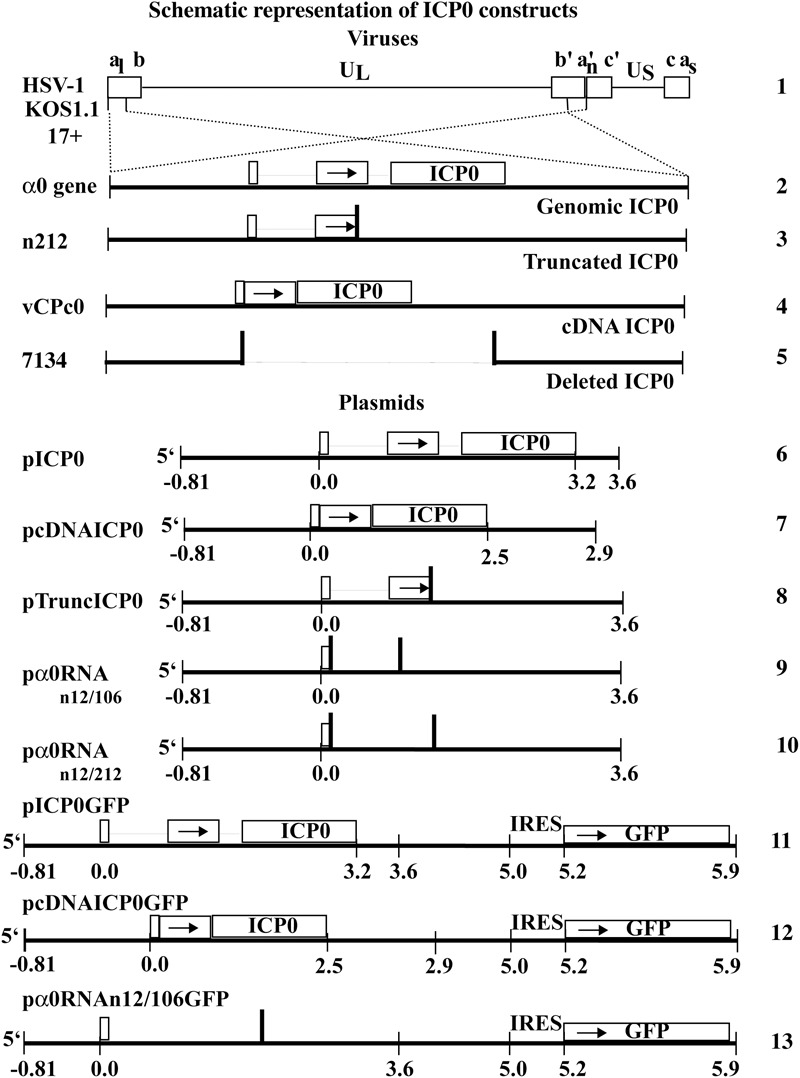
Schematic representation of HSV-1 viral constructs and ICP0 plasmids. HSV-1 (line 1) contains two copies of the α0 gene (line 2), encoding ICP0, in the inverted repeated regions (abc a′b′c′) that flank the unique long region (U_L_) in the HSV-1 genome. The α0 introns and exons are represented by lines and boxes, respectively. The n212 virus generates full-length α0 mRNA but a truncated ICP0 protein. It contains the same α0 sequences as wild-type HSV-1 but there are nonsense mutations in all three reading frames inserted after codon 212 in both copies of ICP0 (line 3). The vCPc0 virus generates cDNA-expressed ICP0 protein from both copies of α0 (line 4). The 7134 virus contains deletions of both copies of α0 and does not produce ICP0 (line 5). The pICP0 plasmid contains the entire genomic ICP0 coding region and flanking sequences (810 bases and 0.4 kB at the 5′ and 3′ ends, respectively) within the pUC8 vector (line 6). The pcDNAICP0 plasmid includes the cDNA copies of ICP0 and flanking sequences from ICP0 coding region (810 bases and 1.1 kB at the 5′ and 3′ ends, respectively) within the pUC19 vector (line 7). pTruncICP0 generates full-length ICP0 mRNA and a truncated protein due to a nonsense mutation after codon 212 (line 8). pα0RNAn12/106 contain nonsense codons at a.a. 12 and 106 (line 9). pα0RNAn12/212 contain nonsense codons at a.a. 12 and 212 (line 10). pICP0GFP contains an internal ribosome entry site (IRES) and the efficient green fluorescent protein (GFP) immediately following the ICP0 gene of pICP0 (line 11). Similarly, pcDNAICP0GFP and pα0RNAn12/106GFP contain the IRES-GFP inserted into pcDNAICP0 and pα0RNAn12/106, respectively (lines 12 and 13).

The trigger of this HSV-dependent apoptosis has been mapped to the HSV-1 α0 gene (Sanfilippo and Blaho, 2006). Initial studies into the viral factors triggering apoptosis in HEp-2 cells utilized the protein synthesis inhibitor, cycloheximide (CHX) (Aubert and Blaho, 1999). The human carcinoma HEp-2 cells infected in the presence of CHX underwent apoptosis, suggesting that at least in these cells, triggering of HSV-dependent apoptosis does not require *de novo* protein synthesis. Further studies using actinomycin D or a recombinant virus lacking all immediate early genes (IE), *d*109, demonstrated that IE gene transcription was required to trigger apoptosis during infection (Sanfilippo et al., 2004a). HSV encodes four immediate early genes, α4, α27, α22, α0. Infections with recombinant viruses containing mutations in the α4 (CgalΔ3), α27 (vBSΔ27), or α22 (R7802) gene maintained the ability to trigger HSV-dependent apoptosis. In contrast, infection with a recombinant virus lacking expression of the α0 gene (7134) failed to induce apoptosis (Sanfilippo and Blaho, 2006). Furthermore, a virus lacking expression of all immediate early genes except for α0, *d*106, maintained the ability to trigger apoptosis. The α0 gene encodes a multifunctional protein of 110 kDa in size, ICP0 (reviewed in Everett, 2000; Hagglund and Roizman, 2004). In HEp-2 cells, infection with a virus containing a nonsense mutation at amino acid 212 of ICP0 (n212), which was therefore, unable to produce full length ICP0 protein, was still capable of inducing apoptosis. Finally, one series of experiments determined that expression of ICP0 from a plasmid outside of an HSV infection induced apoptosis in a dose dependent manner in HEp-2 cells (Sanfilippo and Blaho, 2006). Together, these studies demonstrated that the ability of HSV to trigger apoptosis within infected cells maps to the α0 gene, and that translation of the full-length protein was not required for induction of HSV-dependent apoptosis in HEp-2 cells. The α0 gene is located in the long repeat regions of the HSV-1 genome and is unique compared to other HSV-1 genes in that it contains three exons and two introns (Figure 1, line 2). α0 is also unique because the spliced introns remain stable in the cytoplasm and accumulate in a cell-type-dependent manner (Carter and Roizman, 1996). In this study, we go on to further investigate the nature of α0’s pro-apoptotic activities in HEp-2 cells.

We also expanded our analysis to investigate the role of α0 and ICP0 in apoptosis in primate kidney Vero cells, which have been previously been shown to possess different requirements for HSV-dependent apoptosis compared to HEp-2 cells (Nguyen et al., 2005). Vero cells undergo HSV-dependent apoptosis when infected with the vBSΔ27-mutant virus, but, unlike HEp-2 cells, fail to undergo apoptosis when infected with wild-type HSV in the presence of cycloheximide. In fact, cycloheximide treatment at or before 3 hpi suppressed the apoptosis induced by vBSΔ27 infection. This data led to the conclusion that as opposed to HEp-2 cells, Vero cells require *de novo* synthesis of a protein produced by 3 hpi in order to undergo HSV dependent apoptosis. This protein was named the facilitator of apoptosis (FAP), due to its key role in viral apoptosis (Nguyen et al., 2005). FAP could be either a cellular protein produced prior to 3 hpi, or one of the immediate early proteins. In this study we explore whether the ICP0 protein itself can facilitate viral apoptosis during HSV infection in Vero cells.

## Materials and Methods

### Cell Lines and Viruses

Human epithelial (HEp-2) and African green monkey kidney (Vero) cells were obtained from the American Type Culture Collection (Rockville, MD, United States). It has been shown that the HEp-2 strain, which was previously described to be derived from laryngeal carcinoma, is a HeLa contaminant; we have referred to this HeLa-derived strain as HEp-2 (Nguyen et al., 2005). Vero and HEp-2 cells were cultured in Dulbecco’s modified Eagle’s medium (DMEM) and supplemented with 5% fetal bovine serum (FBS). FO6 cells are a Vero derivative, which express the ICP0, ICP4, and ICP27 genes from their respective viral promoters (Samaniego et al., 1998). FO6 cells were grown in DMEM supplemented with 5% FBS containing 400 μg/ml G418 and 300 μg/ml hygromycin. The Vero-derived cell line expressing ICP0 from its viral promotor, L7 (Samaniego et al., 1998), was grown in DMEM supplemented with 5% FBS containing 400 μg/ml G418. HSV-1 strain KOS1.1 (KOS) and HSV-1 strain 17+ (17) are the wild-type strains of HSV-1 used in this study. The HSV-1 mutant, vCPc0, was generously provided by Dr. Saul Silverstein (Columbia University) and has both ICP0 genomic coding regions of 17 replaced by cDNA copies of the ICP0 transcript (Panagiotidis et al., 1997). Vero cells were used for growth and tittering of wild-type HSV-1 strains and of vCPc0. 7134 and n212 viruses were generously provided by Dr. Pricilla Schaffer (Harvard Medical School). 7134 is an ICP0-null virus, which has both copies of ICP0 in KOS replaced by the *E. coli lacZ* gene (Cai and Schaffer, 1989). This virus was propagated and tittered on FO6 cells. The KOS-derived HSV-1 n212 (n212) contains a stop codon in all three reading frames at amino acid 212 of ICP0 (Cai and Schaffer, 1989). This virus was propagated and tittered on L7 cells. All virus titers were determined at 48 h post-infection by standard dilution techniques.

### Viral Infection and Protein Synthesis Inhibition by CHX

HEp-2 cells were seeded in DMEM supplemented with 5% new born calf serum (NBCS) 24 h prior to infection. Cells were infected at a multiplicity of infection (MOI) of 10 and incubated at 37°C for 1 h. Media was aspirated and replaced with 5% NBCS and incubated at 37°C and 5% CO_2_ for 24 h. In experiments designed to inhibit *de novo* protein synthesis, cycloheximide (CHX) was added directly to the medium of monolayers at a final concentration of 10 μg/ml, which has been previously shown to sufficiently block viral protein synthesis in KOS-infected cells (Aubert and Blaho, 1999). One hour prior to infection, CHX is added at 37°C and was maintained in the medium until 24 h post-infection when morphological and biochemical analyses were performed (described below).

### Plasmids

The pSH, pn212, pn12/106, and pn12/212 plasmids were generously provided by Pricilla Schaffer (Harvard Medical School) and were previously described (Cai and Schaffer, 1989). Briefly, pSH includes the entire ICP0 coding region (3.2 kB) as well as the flanking sequences (0.81 kB 5′ and 0.4 kB 3′) in a pUC8 backbone and for clarity in this study is hereafter referred to as pICP0 (Figure 1, line 6). The plasmid expressing ICP0 from a cDNA copy of ICP0 in pUC19, pDS-16 (Panagiotidis et al., 1997), was generously provided by Saul Silverstein (Columbia University) and hereafter is referred to as pcDNAICP0 (Figure 1, line 7). pn212 is a derivative of pICP0 (Cai and Schaffer, 1989) containing a stop codon in all three reading frames at amino acid 212 and therefore generates full-length α0 transcript and a truncated ICP0 protein, referred to as pTruncICP0 (Figure 1, line 8). pα0RNAn12/106 (Figure 1, line 9) and pα0RNAn12/212 (Figure 1, line 10) are related to pTruncICP0 and contain additional stop codons at a.a. 12 and 106 or 212 respectively. In pICP0, pcDNAICP0, pTuncICP0, pα0RNAn12/106, and pα0RNAn12/212, ICP0 synthesis is regulated by the viral α0 promotor, which is contained in the 5′ flanking region.

pICP0GFP simultaneously expresses ICP0 and the efficient green fluorescent protein (GFP) from the same transcript. To create it, an internal ribosome entry site (IRES) and the GFP gene from the pHR′-CMV MCS IRES GFP delta B plasmid (generously obtained from Mary Klotman, Mount Sinai School of Medicine), were cloned directly after the ICP0 3′ flanking region of the pICP0 plasmid (Figure 1, line 11). pcDNAICP0GFP (Figure 1, line 12) and pα0RNAn12/106GFP (Figure 1, line 13) were created by replacing the ICP0 in pICP0GFP with the cDNA and α0RNAn12/106 versions. The BAK plasmid (pBAK) was obtained from Peter Palese (Mount Sinai School of Medicine) and expresses an HA-tagged BAK protein from the CMV promotor. pUC19 was obtained from New England Biolabs. In several previous studies, the amount of apoptosis in DNA-negative, control transfected cells is undetectable (Goodkin et al., 2003; Sanfilippo et al., 2004a,b; Sanfilippo and Blaho, 2006).

### Transfections

HEp-2 and Vero cells were seeded at 2.5 × 10^5^ cells/2 cm^2^-surface area dish in DMEM containing 5% FBS and transfected 24 h later (approximately 80% confluence) using Lipofectamine 2000 (Invitrogen) according to the manufacturer’s protocol. Briefly, purified plasmid DNA (0.4 and 1.0 μg, respectively) was diluted in 25 μl DMEM. Lipofectamine 2000 reagent (1.0 and 1.5 μl, respectively) was diluted in 25 μl DMEM. After 5 min, the diluted Lipofectamine was added to the DNA solution, and complexes formed at room temperature for 20 min. The media was aspirated off cell monolayers and replaced with 200 μl DMEM, and 50 μl of the DNA-Lipofectamine complex solution was added to each well. Cells were incubated at 37°C and 5% CO_2_ for 4 to 6 h when the media was aspirated and replaced with DMEM supplemented with 10% FBS and incubated until the time of harvest, as denoted in the text.

### Microscopic Analyses and Quantification of Chromatin Condensation

Infected and transfected cell phenotypes were documented using phase-contrast light microscopy with an inverted fluorescence microscope. Images were obtained using QCapture software. For analysis of chromatin condensation in live cells, the DNA dye, Hoechst 33258 (Sigma), was added to media at a final concentration of 5 μg/ml for at least 1 h at 37°C. Phase-contrast (phase) and fluorescent (Hoechst and/or GFP) images were taken for each well. Merged (overlay) images were generated using Adobe Photoshop CS software. The percentage of nuclei containing condensed chromatin to total cells was determined for triplicate wells and the mean (M) and standard deviation (SD) of apoptotic cells were determined for each treatment. Typically, 100–300 cells were counted for each well. For HEp-2 experiments, raw data from five experiments with identical conditions was pooled and overall M and SD were determined:

*i* = Treatment, *j* = Well

$$\begin{array}{l} M_{i}^{HEp-2}=\left(\frac{\sum CondensedChromatin_{i}}{\sum TotalCells_{i}}\right)\\ SD_{i}^{HEp-2}=\sqrt{\frac{\sum {\left(\frac{CondendedChromatin_{ij}}{TotalCells_{ij}}-M_{i}\right)}^{2}}{TotalCells_{i}}} \end{array}$$

Since Vero experiments were done in a time course, data from each time-point was standardized by dividing the percentage of apoptotic cells for each treatment by the percentage of apoptotic cells in pUC19-transfected cells. The M and SD was calculated from three independent experiments using the following formula:

*i* = Treatment, *j* = Well, *k* = Time Point

$$\begin{array}{l} M_{i}^{Vero}=\frac{\sum \left(\frac{[CondensedChromatin_{ijk}/TotalCells_{ijk}}{\left[M_{k}^{pUC19}\right]}\right)}{TotalWells_{i}}\\ SD_{i}^{Vero}=\sqrt{\frac{\sum \left(\frac{[CondensedChromatin_{ijk}/TotalCells_{ijk}}{\left[M_{k}^{pUC19}\right]}-M_{i}\right)}{TotalWells_{i}}} \end{array}$$

### Cellular Extractions, Denaturing Gel Electrophoresis, and Immunoblotting

Whole cell extracts were obtained as previously described (Nguyen et al., 2007). Briefly, cells were scraped into the media, combined and collected by centrifugation for 5 min at 4°C and 1000 × *g*. For transfection experiments, cells from three identically treated wells were combined for protein analysis. Pellets were washed in cold phosphate buffered saline (PBS) containing the inhibitors: 2 mM phenylmethylsulfonyl fluoride (PMSF), 1% Transylol, 0.1 mM L-1-chloro-3-(4-tosulamido)-4-phenyl-2butanone (TPCK), and 0.01 mM L-1-chloro-3-(4-tosylanmido)-7-aminoheptanonhydrochloride (TLCK). Cells were lysed in RIPA buffer (50 mM Tris-HCl, pH 7.5, 150 mM NaCl, 1% Triton X-100, 1% deoxycholate, 0.1% SDS) containing the inhibitors described above and vortexed for 30 s. Lysates were cleared by centrifugation for 10 m at 4°C and 16,000 × *g*. Protein concentrations were determined using a modified Bradford assay (BioRad) as recommended by the vendor. Equal amounts of cell proteins were separated in denaturing 12% *N,N*′-diallyltartardiamide (DATD)-acrylamide gels and electrically transferred to nitrocellulose membranes in a tank apparatus (BioRad). Membranes were blocked for at least 1 h at room temperature in PBS containing 5% non-fat dry milk (blocking buffer), washed with Tris-buffered saline containing 0.1% Tween-20 (TBS-T), and incubated overnight in primary antibody at 4°C. The mouse monoclonal antibodies anti-PARP (PharMingin), -procaspase-7 (BD Transduction), -ICP0 (Goodwin Institute for Cancer Research, Plantation, FL, United States), -VP22 (Blaho et al., 1994), -ICP27 (Goodwin) and -actin (Sigma) were used at a 1:1000 dilution in Tris-buffered saline containing 0.1% Tween 20 and 0.1% BSA. Mouse anti-HA monoclonal antibody (Southern Biotech) (to screen for HA-tagged BAK protein) was used at a 1:5000 dilution. After washing with TBS-T, membranes were treated with secondary anti-mouse antibodies conjugated with alkaline phosphatase (AP) (Southern Biotech) or with horseradish peroxidase (HRP) (GE Healthcare) at a 1:1000 dilution in blocking buffer. Membranes treated with AP secondary were developed using AP buffer containing 5-bromo-4-chloro-3-indolyl phosphate and 4-nitrobluetetrazolium chloride. Membranes treated with HRP antibodies were immersed in western blotting substrates (Roche) and exposed to Biomax XAR film (Kodak). Complete blots were cut with razor blades and sections were probed using specific, relevant antibodies. Blots were reassembled and scanned in a single run. The resulting digital images were cropped as necessary to create figures.

### Densitometric Analysis

To quantify PARP and procaspase-7 cleavage densitometry of images was performed and analyzed using NIH image software. For PARP cleavage, the mean density (MD) of the cleaved PARP band was divided by the sum of MD cleaved and MD uncleaved PARP bands and expressed in a percentage: *i* = Treatment

$$\%\;\text{PARP}\;{\text{cleavage}}_{i}=100\times \left(\frac{MD_{cleaved_{i}}}{\left[MD_{uncleaved_{i}}+MD_{cleaved_{i}}\right]}\right).$$

Procaspase-7 cleavage was determined by dividing MD procaspase-7 band by the MD of the respective band in the actin loading control. Values were normalized to the pUC19 procaspase-7 to actin ratio to determine percentage of procaspase-7 compared to pUC19 as follows. *i* = Treatment

$$\text{Normalized}\;\%\;\text{procaspase-7}\;\text{Cleavage}=100\times \frac{\left[MD_{casp7_{i}}/MD_{actin_{i}}\right]}{\left[MD_{casp7pUC19}/MD_{actinpUC19}\right]}.$$

### Statistical Analysis

To determine statistical significance, Microsoft Excel was used to perform the Student’s *t*-test on apoptotic morphology data (Figure 3, 5, 6, 8). *p*-Values of 0.05 or lower were considered statistically significant.

## Results

### Introns Are Not Required for ICP0’s Proapoptotic Activity in HEp-2 Cells

Because α0 appeared able to confer its pro-apoptotic activity through an RNA-mediated mechanism (Sanfilippo and Blaho, 2006), we asked whether the two ICP0 introns are necessary for inducing apoptosis in HEp-2 cells. We tested whether cDNA-expressed α0 induced apoptosis upon HSV-1 infection in the presence of CHX. HEp-2 cells were treated with CHX and infected with vCPc0, which is a virus derived from the wild-type HSV-1 strain 17 that generates both copies of ICP0 using the viral promotor and cDNA copies of α0. Infection of HEp-2 cells with both wild-type strains of HSV-1, 17 and KOS, in the presence of CHX were used as positive controls. Infection with wild-type HSV-1 strains and vCPc0 in the absence of CHX and infection with the ICP0-null virus, 7134, in the absence and presence of CHX were used as negative controls. At 24 h post-infection, Hoechst DNA dye was added to the media to allow for visualization of chromatin and cellular and nuclear morphologies were assessed. Following imaging, whole-cell extracts were prepared, separated on a denaturing gel, and probed with anti-PARP, -ICP27, -ICP0 and -VP22 antibodies as described in Section “Materials and Methods.” VP22 detection is a marker for late phase viral replication (Blaho et al., 1994).

**FIGURE 2:**
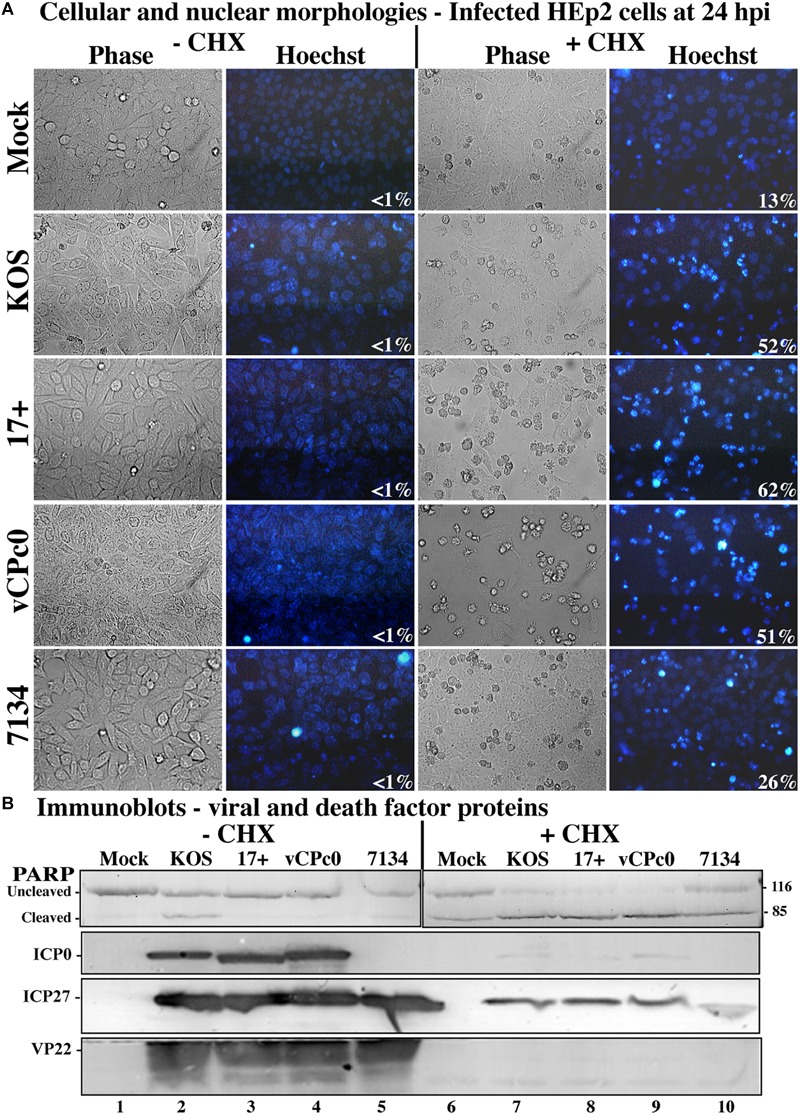
Morphological and biochemical assessment of infected HEp-2 cells at 24 h post-infection. **(A)** HEp-2 cells were infected at MOI of 10 with wild-type HSV-1 (KOS), HSV-1 (17+), and recombinant ICP0 viruses vCPc0 and 7134. Twenty-four hours following post-infection Hoechst DNA dye was added to the media at a concentration of 5 μg/ml to visualize chromatin condensation. Phase contrast and Hoechst-stained images were obtained at 24 h post-infection (40X magnification). Numbers in the lower right corner of Hoechst panels represent the percentage of nuclei displaying chromatin condensation in that treatment. **(B)** Whole-cell extracts were obtained from these cells. Subsequently, the extracted proteins were immunoblotted for PARP, ICP0, ICP27, and VP22. Cropped images of blots were prepared as described in Section “Materials and Methods.”

This experiment was repeated twice and the results from a representative experiment are shown in Figure 2. Phase contrast, Hoechst images, and apoptotic cell percentages are displayed in Figure 2A. The morphological results show that cells infected with wild-type HSV-1 strains, vCPc0 and 7134 in the absence of CHX show enlarged cell size and diffuse cytoplasmic DNA patterns indicative of infectious cytopathic effect (CPE). This morphology results when the HSV-1 virus is regulating cellular machinery and generating HSV-1 virions. Eventually, cells are lysed and viral progeny are released. These treatments show very low levels of apoptosis, since HSV-1 generates anti-apoptotic proteins to prevent programmed cell death to result in infected cells. Cells treated with CHX in the absence of infection show a baseline level of chromatin condensation and membrane blebbing (13%) due to CHX treatment. Cells infected with ICP0-null HSV-1 in the presence of CHX show a baseline level of apoptosis that is slightly increased from CHX treatment alone (26%). This is consistent with previous results (Sanfilippo and Blaho, 2006). KOS and 17 infection in the presence of CHX shows marked increase in apoptotic morphologies (52 and 62%, respectively). Infection with vCPc0 in the presence of CHX shows chromatin condensation and membrane blebbing morphologies at levels (51%) similar to that of KOS.

Additionally, we assessed biochemical markers for apoptosis. When CHX is not present, all wild-type and mutant HSV-1 infections show the presence of VP22, indicating late viral replication and verifying the morphological results (Figure 2B, lanes 2–5). All HSV-1- cells infected in the absence of CHX also generated ICP27 and, with the exception of 7134, ICP0. 7134-infected cells do not generate ICP0 since this is an ICP0-null virus. Conversely, wild-type and mutant infections in the presence of CHX did not synthesize VP22, ICP0, and ICP27 (Figure 2B, lanes 7–10). In the presence of CHX, all HSV-1-infected cells generate low levels of modified ICP27 compared to infection in the absence of CHX. The production of these ICP27 immune reactive triplet forms was previously reported and is likely due to a unique feature of the ICP27 transcript that allows for translation in the presence of CHX (Sanfilippo et al., 2004b). When the apoptotic marker, PARP, was analyzed in this experiment, all wild-type and mutant HSV-1-infected cells without CHX had low levels of PARP cleavage (lanes 2–5). This indicates that little to no apoptosis occurring in these wells, which confirms the morphological results (Figure 2A). CHX-treated cells show baseline PARP cleavage for this treatment (lane 6). 7134-infected cells in the presence of CHX had PARP cleavage similar to CHX-treated cells (compare lane 10 with 6), which indicates that HSV-1 infection without α0 does not induce apoptosis. Whole-cell extracts from KOS- and 17-infected cells in the presence of CHX resulted in nearly complete PARP cleavage (lanes 7–9), indicating high rates of apoptosis and verifying morphological results (Figure 2A). Importantly, vCPc0-infection in the presence of CHX caused PARP cleavage at rates equivalent to KOS-infected cells plus CHX. Together, these morphological and biochemical results indicate that in the absence of protein synthesis intron-less, cDNA-expressed α0 induces apoptosis in infected cells at similar rates to wild-type-infected cells.

**FIGURE 3:**
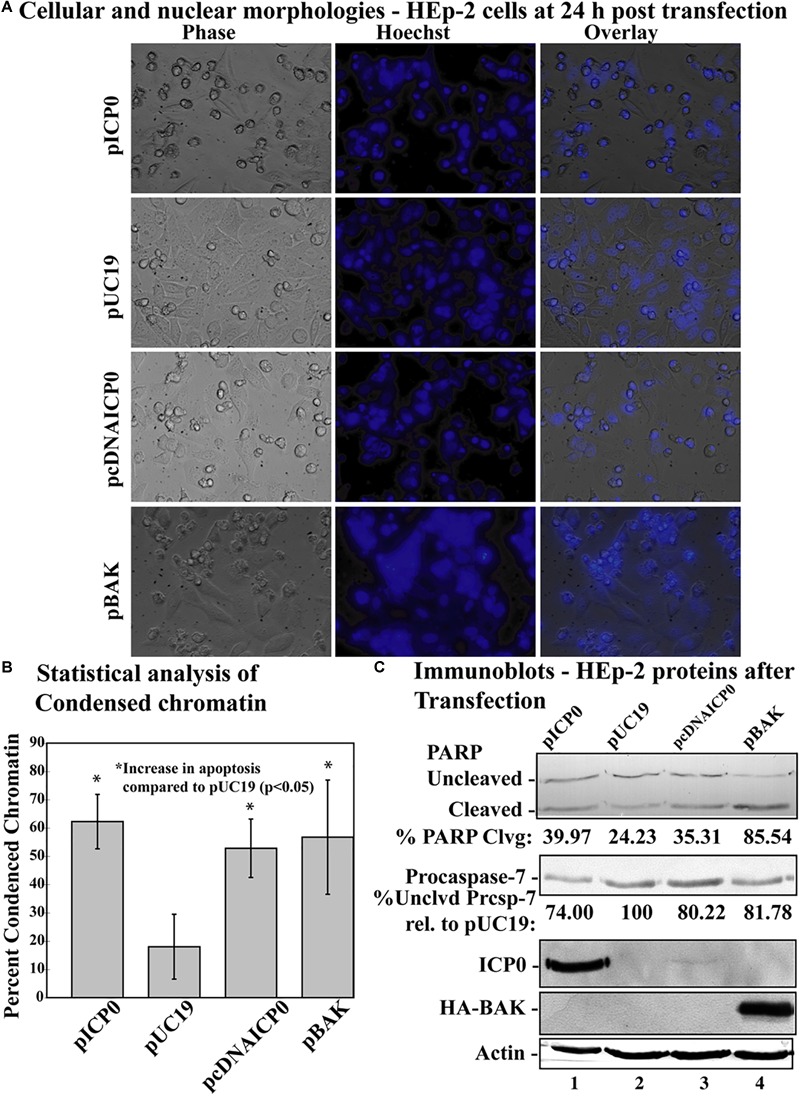
Apoptosis in HEp-2 cells following transfection with pICP0 and pcDNA. **(A)** Cell morphologies of HEp-2 cells transfected with 0.4 μg of pICP0, pcDNAICP0, and pBAK. Twenty-four hours following transfection Hoechst DNA dye was added to the media at a concentration of 5 μg/ml to visualize chromatin condensation. Phase contrast, Hoechst-stained, and overlayed images were captured with a digital camera (40X magnification). Images are representative of a single experiment. **(B)** Statistical analysis of percentages of condensed chromatin for each treatment was conducted using Student’s *t*-test (*p* < 0.05). The results represented in the bar graphs are from five independent experiments performed in triplicate. The mean of the percentage of cells exhibiting chromatin condensation following transfection is graphed. Error bars represent standard deviation for each treatment group. **(C)** Immune reactivities of transfected cells from triplicate wells that were combined, harvested, separated in a denaturing gel, transferred to nitrocellulose, probed with anti-PARP, -ICP0, -actin, -procaspase-7 and -HA primary antibodies. PARP and procaspase-7 band intensities were quantified using NIH image software, as described in Section “Materials and Methods.” Percent PARP cleavage was calculated as a ratio of the band intensity of cleaved PARP relative to the sum of uncleaved and cleaved bands. For procaspase-7 protein, the band intensity for cells transfected with pUC19 was set to 100% and all other groups are displayed relative to this number. Cropped images of blots were prepared as described in Section “Materials and Methods.”

Previous studies have indicated that transfection with a plasmid expressing ICP0 induces apoptosis in cells (Inman et al., 2001b; Sanfilippo and Blaho, 2006). To test whether α0 introns are needed for induction of apoptosis in this setting, we transfected cells with a plasmid expressing ICP0 from a cDNA copy of the α0 gene (pcDNAICP0) and compared the response to cells transfected with a plasmid expressing ICP0 from the intron-containing, genomic copy of the gene (pICP0). The pUC19 plasmid was used as a negative control. In addition, a plasmid (pBAK) expressing the proapoptotic BCl-2 family member, BAK, which induces the intrinsic apoptotic pathway, was used as a positive control. This experiment was repeated five times in triplicate. At 24 h post-transfection, cellular and nuclear morphologies were assessed and the results from a representative experiment are displayed in Figure 3A. The overall means and standard deviations of chromatin condensation from all five experiments are graphed in Figure 3B. Cells transfected with pcDNAICP0 showed more chromatin condensation (52.8 ± 10.3%) compared to pUC19 transfected cells (18 ± 11.4%). This difference was deemed to be statistically significant (*p* < 0.05) using Student’s *t*-test. Cells transfected with positive control plasmids, pICP0 and pBAK, showed levels near and above that for pcDNAICP0, respectively.

Previously, we have demonstrated that procaspase-7 is cleaved and activated during HSV-dependent apoptosis (Kraft et al., 2006); we used immunoblots for this and PARP as markers for apoptosis (Figure 3C). Immunoblotting was also used to detect ICP0 and BAK produced in transfected cells. Transfection with pcDNAICP0 led to an increase in PARP cleavage (35.31%) and a decrease in procaspase-7 protein (80.22%) from pUC19 (24.23% PARP cleavage and 100% procaspase-7 protein). It is of note that the magnitude of biochemical markers for apoptosis in these groups was less than the magnitude of the difference in apoptotic morphologies (Figure 3B). Because we are looking at whole cell lysates from both transfected and non-transfected cells, some of the differences in protein levels can be more difficult to detect, than distinctions in morphologies that are quantitated on a per cell basis. Although pcDNAICP0 produces less ICP0 protein than pICP0 as seen in the ICP0 blot (Figure 3B, compare lane 3 with 1), pcDNAICP0-transfected cells show increased levels of apoptosis from the pUC19 baseline. Based on the results presented in Figure 2, 3, we conclude that the α0 introns are not required for the apoptotic activity of ICP0.

### Apoptosis Is Cell-Autonomous in ICP0-Transfected HEp-2 Cells

**FIGURE 4:**
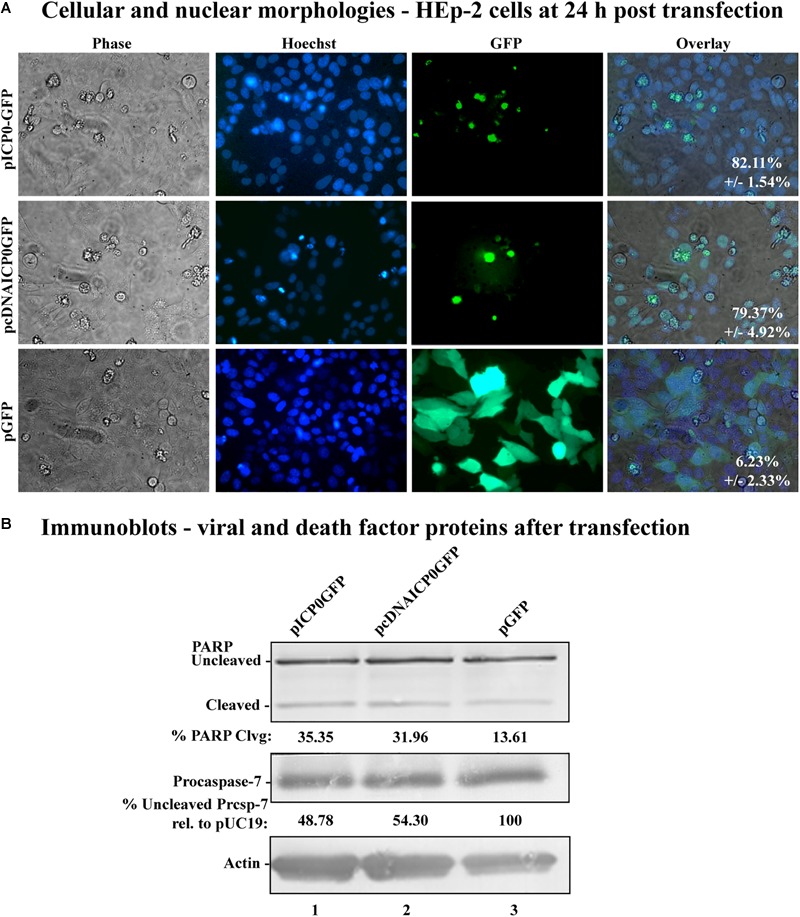
Morphological and biochemical assessment of transfected HEp-2 cells at 24 h post-transfection. **(A)** Cell morphologies of HEp-2 cells transfected with 0.4 μg of pICP0GFP, pcDNAICP0GFP, or pGFP. Fluorescence images were obtained to identify Hoechst DNA staining (Hoechst) and the presence of the GFP. Phase contrast, Hoechst-stained, and GFP images were layered to obtain a merged image (overlay). The numbers inside each frame refers to the percentage of GFP positive cells exhibiting condensed chromatin. **(B)** Whole-cell extracts of HEp-2 cells transfected with pICP0GFP, pcDNAICP0GFP, and a GFP expressing control plasmid (pGFP) were obtained from triplicate wells, separated in a denaturing gel, transferred to nitrocellulose, and probed with anti-PARP, -procaspase-7 and -actin antibodies. PARP and procaspase-7 band intensities were quantified using NIH image software, as described in Section “Materials and Methods.” Percent PARP cleavage was calculated as a ratio of the band intensity of cleaved PARP relative to the sum of uncleaved and cleaved bands. For procaspase-7 protein, the band intensity for cells transfected with pUC19 was set to 100% and all other groups are displayed relative to this number. Cropped images of blots were prepared as described in Section “Materials and Methods.”

The apoptosis induction by α0 transfection could either be explained by ICP0 expression directly causing apoptosis in transfected cells or indirectly causing apoptosis in nearby non-expressing cells via the secretion of a pro-apoptotic factor. To distinguish between these possibilities, we constructed plasmids expressing either the full-length ICP0 open reading frame or the cDNA version, containing GFP driven from an adjacent IRES, termed pICP0GFP and pcDNAICP0GFP, respectively (Figure 1, lines 11 and 12). Transfecting HEp-2 cells with these GFP-expressing constructs allowed us to identify the cells expressing ICP0 and assess their morphologies. HEp-2 cells were transfected with control GFP-expressing plasmid (pGFP), pcDNAICP0GFP, or pICP0GFP. At 24 h following transfection, GFP fluorescence, stained chromatin, and morphological changes were assessed using phase contrast and fluorescence microscopy (Figure 4A). Green fluorescence indicative of GFP expression was evident in pGFP-, pcDNAICP0GFP-, and pICP0GFP-transfected cells. The majority of the GFP positive cells from the pGFP transfection displayed a cobblestone morphology and their chromatin was evenly distributed throughout the nuclei, indicative of a healthy cell monolayer, and similar to the morphologies of the surrounding non-transfected cells. In contrast, the majority of the GFP-positive cells from the pcDNAICP0GFP- and pICP0GFP-transfected wells were smaller, exhibited membrane blebbing, and displayed smaller, more brightly stained nuclei than pGFP-transfected cells. These phenotypes are indicative of cells undergoing apoptosis. We quantitated the percentage of GFP-positive cells displaying apoptotic morphologies to surrounding non-expressing cells in 12 individual wells. 82.11 ± 1.54% of the cells transfected with pICP0GFP and 79.37 ± 4.92% of pcDNAICP0GFP-transfected cells were apoptotic, while only 6.23 ± 2.33% of the cells transfected with pGFP were apoptotic. Cell lysates from transfected cells were assessed for the biochemical features of apoptosis via immunoblotting. pICP0GFP- and pcDNAICP0GFP-tranfected cells showed more PARP and caspase-7 cleavage than control pGFP (Figure 4B, compare lanes 1 and 2 with 3). These results indicate ICP0 expression leads to apoptosis in a cell autonomous manner in the HEp-2 cells.

### Full-Length ICP0 Protein Is Not Required for Apoptosis Induction in HEp-2 Cells

**FIGURE 5:**
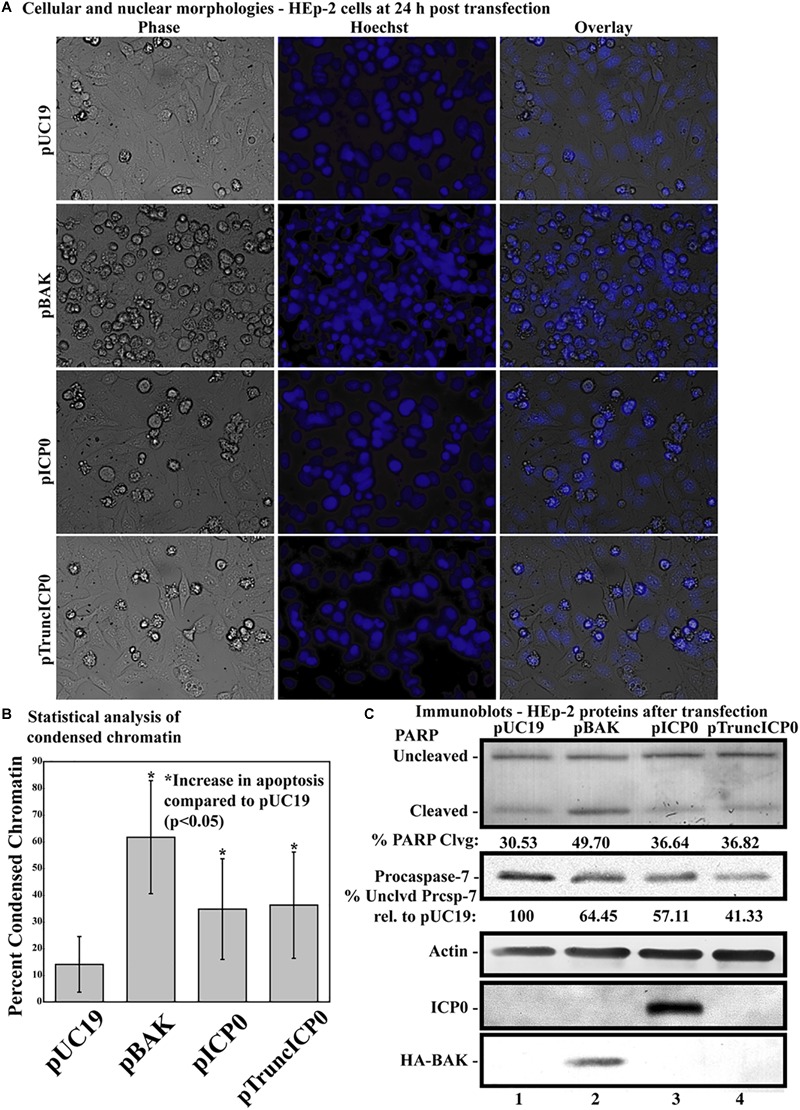
Apoptosis in HEp-2 cells following transfection with pICP0 and pTruncICP0. **(A)** Cell morphologies of HEp-2 cells transfected with 0.4 μg of pUC19, pBAK, pICP0, or pTruncICP0. Phase contrast, Hoechst and overlay images were captured with a digital camera (40X magnification). Images are representative of a single experiment. **(B)** Statistical analysis of percentages of condensed chromatin for each treatment was conducted using Student’s *t*-test (*p* < 0.05). The results represented in the bar graphs are from five independent experiments performed in triplicate. The mean of the percentage of cells exhibiting chromatin condensation following transfection is graphed. Error bars represent the standard deviation for each treatment group. **(C)** Immune reactivities of transfected cells from triplicate wells that were combined, harvested, separated in a denaturing gel, transferred to nitrocellulose, probed with anti-PARP, -procaspase-7, -ICP0, -actin, and -HA (to recognize HA-tagged BAK protein) primary antibodies. PARP and procaspase-7 band intensities were quantified using NIH image software, as described in Section “Materials and Methods.” Percent PARP cleavage was calculated as a ratio of the band intensity of cleaved PARP relative to the sum of uncleaved and cleaved bands. For procaspase-7 protein, the band intensity for cells transfected with pUC19 was set to 100% and all other groups are displayed relative to this number. Cropped images of blots were prepared as described in Section “Materials and Methods.”

Previous studies showed that HSV-1 infected cells undergo HSV-dependent apoptosis when protein synthesis is inhibited and when both copies of genomic ICP0 contain a stop codon (Sanfilippo and Blaho, 2006). This finding suggests that the ICP0 RNA is a proapoptotic stimulus in infected HEp-2 cells. The next investigation was to determine whether partial ICP0 protein synthesis could facilitate α0’s proapoptotic activity in HEp-2 cells. HEp-2 cells were transfected with a plasmid expressing a mutant of ICP0 with a nonsense mutation after codon 212, pTruncICP0 (Figure 1, line 8). The vector pUC19 was transfected as a negative control. The pBAK and pUC19 plasmids were used as positive and negative controls, respectively. At 24 h post-transfection, cellular and nuclear morphologies were assessed. This experiment was repeated in five times in triplicate. Phase contrast and Hoechst images from a representative experiment are displayed in Figure 5A. The overall means and standard deviations of chromatin condensation from all five experiments are graphed in Figure 5B. The cumulative results (Figure 5) were as follows. 14.07 ± 10.7% of the pUC19-transfected cells showed chromatin condensation, which indicates a baseline level of apoptosis due to transfection. 61.7 ± 22% of the pBAK-transfected cells exhibited chromatin condensation. Transfection of HEp-2 cells with pICP0 showed a statistically significant increased in chromatin condensation (34.8 ± 19.4%) compared to pUC19-transfected cells. Cells transfected with pTruncICP0 showed comparable amounts of chromatin condensation (36.9 ± 19.4%) to pICP0-transfected cells.

Additionally, we assessed the biochemical markers of apoptosis by immunoblotting (Figure 5C). Procaspase-7 and the downstream caspase substrate PARP were used as markers for apoptosis. pUC19 transfection led to 30.53% PARP cleavage, showing background levels of apoptosis due to transfection protocols. We set the levels of procaspase-7 protein present in this group to 100%. pBAK-transfected cells displayed 49.7% PARP cleavage and 64.45% procaspase-7 protein relative to pUC19. This is consistent with the majority of cells undergoing apoptosis as based on the morphological assessment above. pICP0- and pTruncICP0-transfected cells displayed similar levels of PARP cleavage (36.64 and 36.82%, respectively) and procaspase-7 protein (57.11 and 41.33%, respectively). The above morphological and biochemical results show that full-length ICP0 protein is not necessary for ICP0-induced apoptosis in transfected cells. These findings suggest other properties of the α0 gene may affect apoptosis induction in HEp-2 cells.

### ICP0 RNA Is Sufficient to Induce Apoptosis in HEp-2 Cells

**FIGURE 6:**
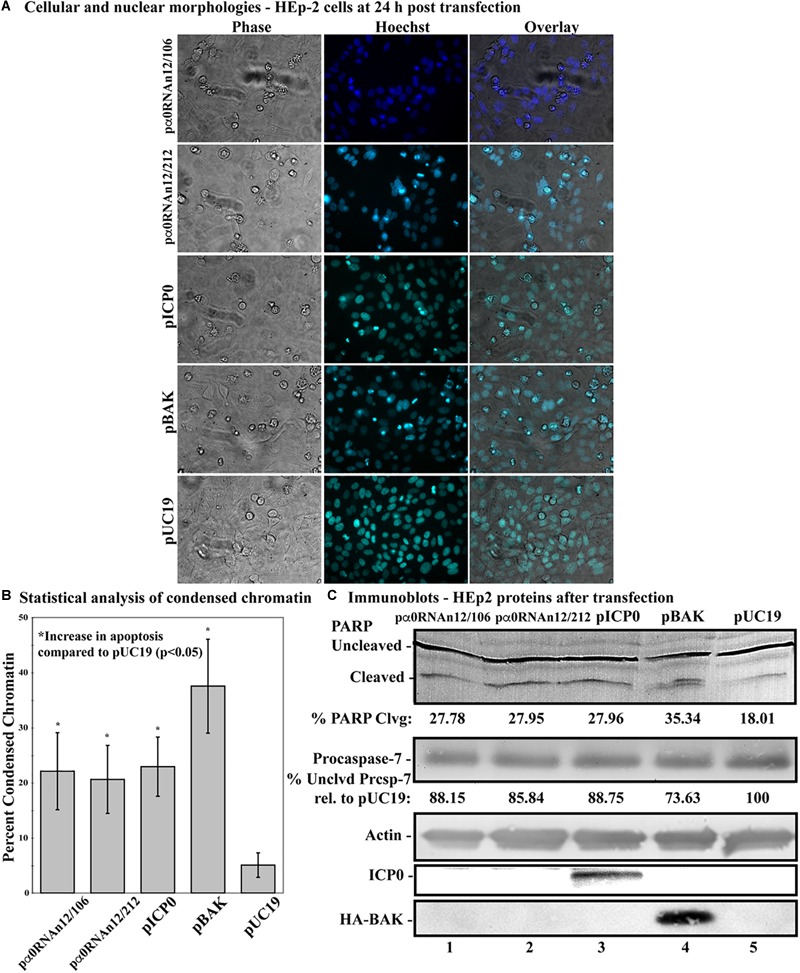
Apoptosis in HEp-2 cells following transfection with pα0RNAn12/106, and pα0RNAn12/212. **(A)** Cell morphologies of HEp-2 cells transfected with 0.4 μg of pα0RNAn12/106, pα0RNAn12/212, pICP0, pBAK, or pUC19. Phase contrast, Hoechst-stained, and overlayed images were captured with a digital camera (40X magnification). Images are representative of a single triplicate experiment. **(B)** Statistical analysis of percentages of condensed chromatin for each treatment was conducted using Student’s *t*-test (*p* < 0.05). The results represented in the bar graphs are from five independent experiments performed in triplicate. The mean of the percentage of cells exhibiting chromatin condensation following transfection is graphed. Error bars represent standard deviation for each treatment group. **(C)** Immune reactivities of transfected cells from triplicate wells that were combined, harvested, separated in a denaturing gel, transferred to nitrocellulose, probed with anti-PARP, -procaspase-7, -ICP0, -actin, and -HA primary antibodies. PARP and procaspase-7 band intensities were quantified using NIH image software, as described in Section “Materials and Methods.” Percent PARP cleavage was calculated as a ratio of the band intensity of cleaved PARP relative to the sum of uncleaved and cleaved bands. For procaspase-7 protein, the band intensity for cells transfected with pUC19 was set to 100% and all other groups are displayed relative to this number. Cropped images of blots were prepared as described in Section “Materials and Methods.”

We next investigated the ability of ICP0 gene expression to induce apoptosis in the absence of ICP0 protein production in transfected cells HEp-2 cells. HEp-2 cells were transfected either with a plasmid expressing genomic α0 with two stop mutations after codons 12 and 106, pα0RNA12/106, or after codons 12 and 212, pα0RNAn12/212 (Figure 1, lines 9 and 10). The vector pUC19 was transfected as a negative control, and pICP0 and pBAK were used as positive controls. At 24 h post-transfection, cellular and nuclear morphologies were assessed. This experiment was repeated in triplicate wells two times. Phase contrast and Hoechst images from a representative experiment are displayed in Figure 6A. The cumulative means and standard deviations of chromatin condensation from both experiments are graphed in Figure 6B. 5.1 ± 2.2% of the pUC19-transfected cells showed chromatin condensation. 37.5 ± 8.5% of the pBAK-transfected cells exhibited chromatin condensation. Transfection of HEp-2 cells with pICP0 showed a statistically significant increased in chromatin condensation (22.9 ± 5.4%) compared to pUC19-transfected cells. Cells transfected with pα0RNAn12/106 and pα0RNAn12/212 showed comparable amounts of chromatin condensation to pICP0-transfected cells (22.1 ± 7.0 and 20.6 ± 6.2%, respectively).

PARP and procaspase-7 cleavage were assessed for biochemical indication of apoptosis using immunoblotting (Figure 6C). pUC19 transfection leads to 18.01% PARP cleavage, showing background levels of apoptosis due to transfection protocols, and again we set the levels of procaspase-7 protein in this group to 100%. pBAK-transfected cells displayed 35.34% PARP cleavage and 73.63% procaspase-7 protein relative to pUC19, which is consistent with a significant portion of cells undergoing apoptosis as based on the morphological assessment above. pICP0-, pα0RNAn12/106- and pα0RNAn12/212-transfected cells displayed similar levels of PARP cleavage (27.78, 27.95, and 27.96, respectively) and procaspase-7 protein (88.15, 85.84, and 88.75, respectively).

Together, the above morphological and biochemical findings show that ICP0 protein is not necessary for ICP0-induced apoptosis in transfected cells. These results support previous findings which suggest that ICP0 RNA is responsible for HSV-1-induced apoptosis in HEp-2 cells.

### ICP0 Protein Is Not Required for Autonomous Proapoptotic Activity in HEp-2 Cells

**FIGURE 7:**
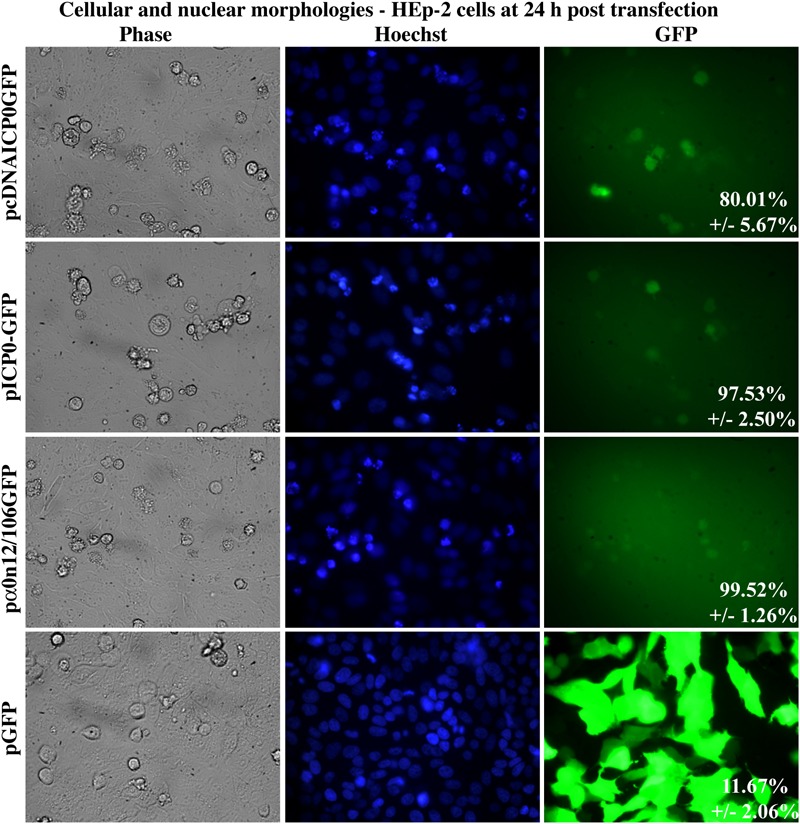
Apoptosis in HEp-2 cells following transfection with pα0RNAn12/106GFP. Cell morphologies of HEp-2 cells transfected with 0.4 μg of pcDNAICP0GFP, pICP0GFP, pα0RNAn12/106GFP, or pGFP. Phase contrast, Hoechst-stained, and overlayed images were captured with a digital camera (40X magnification). Images are representative of a single experiment and numbers inside the overlay represent the mean and standard deviation of the percentage of GFP positive cells displaying condensed chromatin in the given triplicate experiment.

Results above (Figure 4) indicate that transfection of the wild-type ICP0 gene causes apoptosis in a cell autonomous manner. To determine whether this was also true for α0 RNA expression alone the ICP0 open reading frame of pICP0GFP was replaced with α0 with two stop mutations after codons 12 and 106, pα0RNA12/106, termed pα0RNAn12/106GFP. This plasmid was transfected into HEp-2 cells; pcDNAICP0GFP, pICP0, and pGFP were transfected into cells as controls. At 24 h following transfection, GFP fluorescence, chromatin condensation, and morphological changes were assessed using phase and fluorescence microscopy (Figure 7). Green fluorescence indicative of GFP expression was evident in all transfected cells. The majority of the GFP positive cells from the pGFP transfection displayed cobblestone morphology and their chromatin was evenly distributed throughout the nuclei, indicative of a healthy cell monolayer, and similar to the morphologies of the surrounding non-transfected cells. In contrast, the majority of the GFP-positive cells from the pICP0GFP-, pcDNAICP0GFP-, and pα0RNAn12/106GFP-transfected wells were smaller, exhibited membrane blebbing, and displayed smaller, more brightly stained nuclei than surrounding non-transfected cells. These phenotypes are indicative of cells undergoing apoptosis. We quantified the percentage of GFP-positive cells displaying apoptotic morphologies to surrounding non-GFP-expressing cells in 12 individual wells. 80.01 ± 5.67, 97.53 ± 2.50, and 99.52 ± 1.26% of the cells transfected with pcDNAICP0GFP, pICP0GFP, pα0RNAn12/106GFP were apoptotic, while only 11.67 ± 2.06% of the cells transfected with pGFP were apoptotic. Together, these results indicate that ICP0 protein is not required for the autonomous proapoptotic activity of α0 in HEp-2 cells.

### A Nonsense Mutation at Amino Acid 212 Abrogates Apoptosis in Vero Cells

**FIGURE 8:**
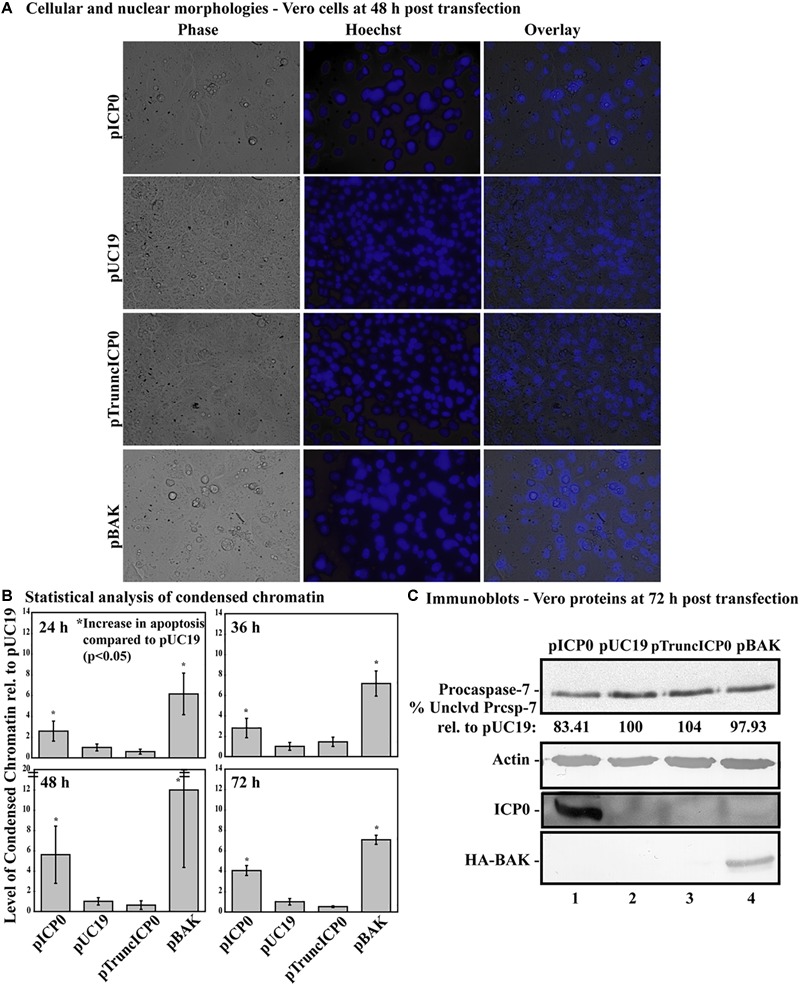
Vero cells do not undergo apoptosis following transfection with pTruncICP0. Vero cells were transfected with 1.0 μg of pICP0, pUC19, pTruncICP0, or pBAK and Hoechst DNA dye was added to the media at 23 h post-transfection. At 24, 36, 48, and 72 h post-transfection, phase contrast and fluorescence microscopy was used to visualize cellular and nuclear morphologies. **(A)** Phase contrast, Hoechst-stained, and overlay images were obtained at 48 h post-transfection (40X magnification). **(B)** The percentage of chromatin condensation at 24, 36, 48, and 72 h in triplicate wells was calculated for four independent experiments. The mean chromatin condensation percentage was calculated for each time point. The percentage of condensed chromatin in pUC19 treated cells was set to 1 for each time point and other groups are presented as a relative value compared to pUC19. The mean relative chromatin condensation is graphed. Error bars represent the standard deviation. Statistical analysis of percentages of condensed chromatin for each treatment and time point was conducted using Student’s *t*-test. **(C)** Triplicate wells were combined at 72 h post-transfection and whole-cell extracts were obtained, separated in a denaturing gel, transferred to nitrocellulose, probed with anti-procaspase-7, -actin, -ICP0, -HA primary antibodies. The intensities of procaspase-7 bands were quantified using NIH image, as described in Section “Materials and Methods.” For procaspase-7 protein, the band intensity for cells transfected with pUC19 was set to 100% and all other groups are displayed relative to this number. Cropped images of blots were prepared as described in Section “Materials and Methods.”

Previously, we determined while infected HEp-2 cells can undergo HSV-dependent apoptosis in the absence of protein expression, infected Vero cells require expression of a protein facilitator for HSV-dependent apoptosis (Nguyen et al., 2005). Given that some form of infected cell protein synthesis was needed for apoptosis in infected Vero cells, it was possible that it was the ICP0 protein itself that facilitates this viral apoptosis. To determine if full-length ICP0 protein was necessary for the α0 induced apoptosis, Vero cells were transfected with pTruncICP0. The pUC19 plasmid was used as a negative control and pICP0 and pBAK were used as positive controls. At 24, 36, 48, and 72 h post-transfection, cellular, and nuclear morphologies were assessed and the results from a representative experiment at 48 h post-transfection are shown in Figure 8A. Apoptotic percentages were determined by dividing the number of apoptotic cells by the total number of cells per image. To account for any minor variations in background levels of apoptosis throughout the timecourse, each treatment was divided by the apoptotic percentage of the pUC19 well for the given time point to calculate the fold increase in apoptosis from pUC19. The means and standard deviations for each timepoint were determined and displayed graphically in Figure 8B. pUC19-transfected cells showed little chromatin condensation (1.66 ± 0.92%). This indicates that the baseline level of apoptosis due to transfection for Vero cells is very low. The pBAK-transfected cells displayed chromatin condensation that was 6–12 fold higher than that of pUC19. Transfection of Vero cells with pICP0 showed a statistically significant increase in chromatin condensation (2.6–5.6 times) compared to pUC19. Cells transfected with pTruncICP0 showed low levels of chromatin condensation (0.6–1.4) relative to pUC19.

At 72 h post-transfection, whole-cell extracts from transfected cells were analyzed for apoptotic proteins, ICP0 and BAK using immunoblotting (Figure 8C). Lysates from pBAK-transfected cells exhibited detectable decreases in procaspase-7 protein (97.93%) compared to pUC19. These findings show that Vero cells are more resistant to BAK-induced apoptosis than HEp-2 cells. Transfection with pICP0 led to decreased levels of procaspase-7 (83.41%) compared to pUC19 (100%). pTruncICP0 transfection displayed little to no reduction of procaspase-7 levels (104%) compared to pUC19. These results demonstrate that full-length ICP0 protein is necessary for ICP0-induced apoptosis in transfected Vero cells. Based on these findings, we conclude that the facilitator protein must be either full-length ICP0 or a cellular protein that is dependent on ICP0.

### Apoptosis Is Cell-Autonomous in ICP0-Transfected Vero Cells

**FIGURE 9:**
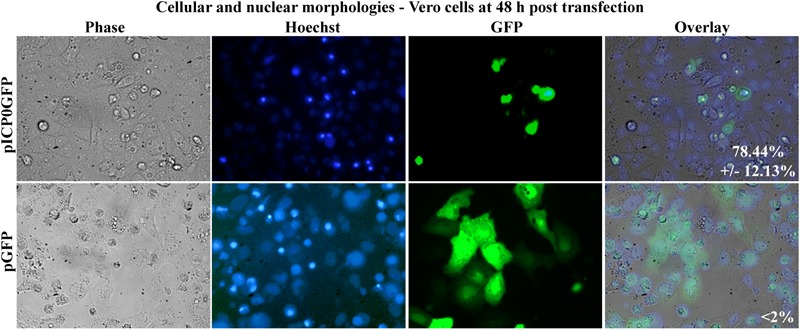
Apoptosis in Vero cells following transfection with pICP0GFP. Cell morphologies of Vero cells transfected with 0.4 μg of pICP0GFP or pGFP. Phase contrast, Hoechst-stained, and overlayed images were captured with a digital camera (40X magnification). Images are representative of a single experiment and numbers inside the overlay represent the mean and standard deviation of the percentage of GFP positive cells displaying condensed chromatin quantified in the given triplicate experiment.

In this final study, we assessed whether Vero cells expressing ICP0 were undergoing apoptosis themselves or if they were releasing proapoptotic factors causing death in surrounding non-transfected cells. To differentiate between these possibilities, Vero cells were transfected with pICP0GFP so we could identify the cells expressing ICP0 and assess their morphologies. Transfection with pGFP was used as a negative control. At 48 h post-transfection, Hoechst DNA dye was added to the media to allow for visualization of chromatin. Subsequent GFP fluorescence, chromatin condensation and morphological changes were assessed using phase and fluorescence microscopy (Figure 9). The GFP positive cells from the pGFP transfection were flat and well spread out, similar to surrounding non-transfected cells. Additionally, they displayed an evenly distributed nuclear Hoechst staining pattern, which is typical of healthy cells. The GFP-positive cells from the pICP0GFP-transfected wells displayed phenotypes indicative of cells undergoing apoptosis. We quantified the percentage of GFP-positive cells displaying apoptotic morphologies compared to surrounding non-expressing cells. 78.44 ± 12.13% of the cells transfected with pICP0GFP were apoptotic. This result indicates that the cells are undergoing ICP0-dependent apoptosis do it in a cell autonomous manner. Thus, if a cellular protein is also involved in the apoptotic process in Vero cells, it does not likely migrate to surrounding cells.

## Discussion

A link between apoptosis and the latency of HSV is supported by many studies from multiple research groups. Therefore, studies of apoptosis in HSV infected cells is likely to provide insight into the establishment, maintenance, and reactivation from latency. Our previous studies have identified the α0 gene as the viral trigger of apoptosis during HSV infections. In this study, we set out to address several key unknowns. The first was focused on the structure of the α0 gene in apoptosis induction in HEp-2 cells. The second involved whether this process occurred in a cell autonomous manner. Finally, we investigated the role of ICP0 protein in apoptosis in Vero cells. Our key findings may be summarized as follows.

ICP0 is sufficient to serve as the herpes apoptosis facilitator (HDAP) in Vero cells. This does not exclude the possibility that other cellular proteins may also be involved. This finding is consistent with previous studies (Inman et al., 2001b) and may be related to the noted ICP0 toxicity in viral gene therapy vectors (Samaniego et al., 1998). The key question remains about the basis of the difference between HEp-2 and Vero cells in the requirements for apoptosis induction. HEp-2 cells are transformed cells and fail to express p53, due to the presence of the human papillomavirus E6 protein (Ogura et al., 1993), which has been previously shown to influence HSV-dependent apoptosis (Nguyen et al., 2007). Vero cells are immortalized, but not transformed; this distinction appears to imply that the pro-apoptotic activity of ICP0 may be affected by p53 status. Previous reports have shown that ICP0 can mediate the ubiquitination of p53 by acting as a RING finger ubiquitin E3 ligase (Boutell and Everett, 2003). Interestingly, a recent study described a reduced replication of HSV-1 in p53 deficient cells. They also reported that the ICP0 protein itself could be degraded in a p53-dependent manner in cells and that this was inhibited during a wild-type infection by ICP22 (Maruzuru et al., 2013), suggesting that a complex interplay between ICP0 and p53 may exist during HSV-1 infection.

α0-Dependent apoptosis likely plays a role during HSV reactivation from latency. ICP0 is essential for viral replication upon reactivation from HSV latency (Leib et al., 1989b). It is now recognized that VP16 is key to reactivation (Kim et al., 2012; Sawtell and Thompson, 2016). α0 is the first viral gene transcribed by VP16 (Harris-Hamilton and Bachenheimer, 1985). That both α0 RNA and ICP0 protein are proapoptotic in certain cells, raises the possibility that neuronal apoptosis occurs during reactivation. This might explain previous observations of apoptosis in herpes encephalitis (DeBiasi et al., 2002).

Although the LATs and miRNA of HSV are the only HSV gene products found to be abundantly expressed during latency, previous studies have detected low levels of α0 transcripts during HSV latency (Chen et al., 2002; Pesola et al., 2005; Maillet et al., 2006). A recent study by Raja and colleagues indicated that interruption of ICP0 expression led to reduced association of viral genomes with histones and lower levels of LAT transcripts in a murine latency model (Raja et al., 2016). The authors of this study propose a model in which low levels of ICP0 protein expression contribute to the establishment and/or maintenance of latency by facilitating genome association with heterochromatin and enhancing LAT expression. It is possible that ICP0’s pro-apoptotic functions may also contribute to this newly identified role for ICP0 in establishment and maintenance of latency.

Together, this study has determined that the α0 gene produces two pro-apoptotic gene products during HSV infection. The α0 transcript is sufficient for apoptosis induction in at least one cell line, HEp-2 cells. On the other hand, other cell types, e.g., Vero cells, require both the transcript and protein of the ICP0 open reading frame to trigger HSV-dependent apoptosis. It is important to note that primary human keratinocytes, the first cells that the virus usually interacts with, are susceptible to HSV-dependent apoptosis (Pradhan and Nguyen, 2013). Further investigation into α0-dependent apoptosis may allow for an improved understanding of the control of both latent and lytic HSV-1 infections. The ICP0 protein is necessary for viral replication upon reactivation (Leib et al., 1989b). Additionally, a recent study has reported that disruption of ICP0 expression led to reduced LAT transcript levels (Raja et al., 2016). Thus, both apoptosis regulation and α0 gene expression have been implicated in latency regulation. Investigators seeking to understanding the role of ICP0 in HSV latency most now consider the potential that both α0 RNA and protein may trigger apoptosis as part of the reactivation process.

## Author Contributions

MN and JB designed, supervised, and interpreted the experiments and finalized the figures and manuscript. EG and KP performed the experiments as research coordinators. EG organized the original figures and wrote an initial version of the manuscript.

## Conflict of Interest Statement

The authors declare that the research was conducted in the absence of any commercial or financial relationships that could be construed as a potential conflict of interest.
